# PB1 S524G mutation of wild bird-origin H3N8 influenza A virus enhances virulence and fitness for transmission in mammals

**DOI:** 10.1080/22221751.2021.1912644

**Published:** 2021-06-06

**Authors:** Xinghai Zhang, Yuanguo Li, Song Jin, Yiming Zhang, Leiyun Sun, Xinyu Hu, Menglin Zhao, Fangxu Li, Tiecheng Wang, Weiyang Sun, Na Feng, Hongmei Wang, Hongbin He, Yongkun Zhao, Songtao Yang, Xianzhu Xia, Yuwei Gao

**Affiliations:** a Department of Veterinary Preventive Medicine, College of Veterinary Medicine, Jilin University, Changchun, People’s Republic of China; b Key Laboratory of Jilin Province for Zoonosis Prevention and Control, Institute of Military Veterinary Medicine, Academy of Military Medical Sciences, Changchun, People’s Republic of China; c Jiangsu Co-innovation Center for Prevention and Control of Important Animal Infectious Diseases and Zoonoses, Yangzhou, People’s Republic of China; d Ruminant Diseases Research Center, College of Life Sciences, Shandong Normal University, Jinan, People’s Republic of China

**Keywords:** Wild birds, H3N8 influenza virus, mammals, transmission, molecular basis

## Abstract

Influenza H3N8 viruses have been recovered frequently from wild bird species, including Anseriformes (primarily from migratory ducks) and Charadriiformes (primarily from shorebirds). However, little attention has been given to the transmission ability of H3N8 avian influenza viruses among mammals. Here, we study the potential human health threat and the molecular basis of mammalian transmissibility of H3N8 avian influenza viruses isolated from wild bird reservoirs. We classified eight H3N8 viruses into seven different genotypes based on genomic diversity. Six of eight H3N8 viruses isolated naturally from wild birds have acquired the ability to bind to the human-type receptor. However, the affinity for α-2,6-linked SAs was lower than that for α-2,3-linked SAs. Experiments on guinea pigs demonstrated that three viruses transmitted efficiently to direct-contact guinea pigs without prior adaptation. Notably, one virus transmitted efficiently via respiratory droplets in guinea pigs but not in ferrets. We further found that the PB1 S524G mutation conferred T222 virus airborne transmissibility between ferrets. We also determined that the 524G mutant increased viral pathogenicity slightly in mice compared with the WT (wild type). Based on these results, we elucidated the potential human health threat and molecular basis of mammalian transmissibility of H3N8 influenza viruses. We emphasized the need for continued surveillance of the H3N8 influenza viruses circulating in birds.

## Introduction

Influenza A viruses belonging to the family Orthomyxoviridae contain eight segments of single-stranded, negative-sense RNA. Because of a higher error rate during replication due to lacking proofreading mechanism, influenza A viruses have rich genetic diversity. Based on the genetic and antigenic variability of their surface proteins HA and NA, influenza viruses are classified into different antigenic subtypes such as 18 hemagglutinin (HA) and 11 neuraminidase (NA) [1]. Wild birds form a large gene pool for influenza A viruses in nature, such that 16 HA and 9 NA subtypes can be found in this reservoir [2].

The wild bird reservoir plays an essential role in the emergence, evolution, maintenance and spread of zoonotic influenza viruses. For example, the highly pathogenic avian influenza A (H5N1) virus, which has spilled over repeatedly to humans since its first report in 1996, did spread all over the world via bird migration [2]. A novel avian influenza virus H7N9 has caused serious human infections across China since March 2013, and this virus is produced by genetic reassortment of wild bird avian influenza virus and poultry avian influenza virus [3]. Most recently, emerging H5Nx influenza viruses including H5N6 and H5N8 virus are circulating in poultry and wild birds and causing economic loss to animal production. Of note, H5N6 virus has crossed the species barrier and caused multiple human infections in China and put a threat to human health. As of 15 January 2021, 27 laboratory-confirmed human infection cases with influenza A (H5N6) virus have been reported to WHO from China [4,5]. Once these zoonotic influenza viruses acquire the ability to be transmitted from human to human efficiently, a pandemic would result, endangering humans’ lives globally. Three subtypes (H1N1, H2N2, and H3N2) of influenza viruses except the 1918-19 H1N1 influenza virus, of which some genes emerged from the wild aquatic bird reservoir have caused influenza pandemics in humans [6].

Influenza H3N8 viruses were recovered from a range of wild bird species including Anseriformes (primarily from migratory ducks) and Charadriiformes (primarily from shorebirds) species. Furthermore, sporadic cross-species transmission events of the H3N8 influenza virus have been reported for various species such as pigs, dogs, horses, seals and donkeys [7–11]. Although infected birds remain healthy or show only mild disease, H3N8 viruses may cause severe respiratory disease in mammal host even lead to death [7]. On a special note, the H3N8 avian influenza viruses have established stable lineages in dogs and horses. Although sporadic cases of H3N8 avian IAV infection in humans have not been reported, a prior study suggested that the seal H3N8 virus could be transmitted through respiratory droplets in the ferrets model that is widely used to evaluate the pandemic potential of influenza viruses in humans [12]. The above researches indicate that H3N8 AIVs pose a potential threat to animal and human health and has the potential to trigger the epidemic of virus in the population.

Some experimentally verified molecular bases involved in AIV pathogenicity, receptor binding, and transmission in mammals have been established. Several mutations in HA are associated with changes in viral fitness and transmissibility. T160A (H3 numbering) increased virus binding to α-2,6 sialic acid receptor and enhance transmission capability of H5N1 in guinea pigs [13,14]. Q226L in HA increases virus binding to α-2,6 sialic acid receptor, and enhances contact transmission of H9N2 in ferrets [15,16]. 225E of HA is important for the respiratory droplet transmission in guinea pigs for Eurasian avian-like H1N1 swine influenza virus [17]. Four amino acid substitutions, including H110Y, T160A, Q226L, and G228S in HA, and E627 K in PB2 collaboratively confer airborne transmissible to H5N1 in ferrets [14]. N158D, N224 K, Q226L, and T318I collectively confer respiratory droplet transmission to a reassortant H5 HA/H1N1 virus in ferrets [18].

The amino acid changes E627 K, D701N, and G590S/Q591R in PB2 have been associated with host-range transmission [19–22]. Huihui Kong et al. found 292 V and 627 K in PB2 and 156D in M1 are extremely important for H7N9 virus transmission in guinea pigs [23]. Furthermore, H99Y in PB1 combined with PB2-E627 K and several amino acid substitutions in HA confer airborne transmission to H5N1 viruses [24]. NS1 has been determined as a critical component of the airborne transmission phenotype of H1N1 AI viruses. Wild bird AIVs with the mutation S213P of NS1 fail to transmit in ferrets [25]. Moreover, reassorted H5N1 viruses comprising the NS gene of pH1N1 2009 are transmitted by air among experimentally infected guinea pigs [26]. These studies indicate that the host-range transmission of the influenza virus is a polygenic trait, and more questions such as the role of PB1 in the host-range transmission must be answered before we can fully understand the molecular mechanisms of transmission of AIV in mammals.

Thus, the properties such as diverse hosts, mammalian adaptation phenotype, and airborne transmission make it necessary to perform surveillance and risk analysis of the H3N8 avian influenza virus from wild birds. The stable mammalian transmission ability is one of the preconditions for a novel influenza virus to spread in the crowd. Ferret is a good model to evaluate the mammalian transmission ability of the influenza virus [12]. Here, to understand the potential risk of the H3N8 avian influenza virus spreading into mammals, we studied the genetic characteristics, receptor-binding properties, and the transmission ability of viruses among guinea pigs and ferrets of a panel of avian H3N8 viruses isolated from wild birds. We found that H3N8 virus can be transmitted among mammals via respiratory droplets, and identified the molecular basis of the virus transmission among ferrets. Our findings show that the H3N8 avian influenza viruses in wild birds pose a potential threat to public health, and we need to strengthen the monitoring and research of these viruses in wild birds.

## Materials and methods

### Ethics statements

This study was conducted in strict accordance with the recommendations in the Guide for the Care and Use of Laboratory Animals of the Ministry of Science and Technology of the People’s Republic of China. We conducted all animal experiments under the Institute of Military Veterinary Medicine’s guidance, the Academy of Military Medical Sciences.

### Facility

According to the Guidelines for the Care and Use of Animals in Research, the animal experiments were conducted in the enhanced animal biosafety level 2+ (ABSL 2+) issued by the Institute of Military Veterinary Medicine, Academy of Military Medical Sciences. The animal experiments with live H3N8 viruses were conducted within the animal isolators in the hyper-filtered facility.

### Virus

Two H3N8 virus from a shorebird of the Charadriiformes order and six H3N8 isolates from a bird of the Anseriformes order were isolated during our surveillance from 2013 to 2019 along East Asian-Australasian migratory bird flyway. We isolated avian influenza viruses from cloacal swabs by inoculation of allantoic cavity of ten-day-old embryonated specific-pathogen-free (SPF) eggs. All viruses were then passaged three times by limiting dilution in embryonated chicken eggs. The virus stock titers were determined by inoculating the chicken eggs with 10-fold serial dilutions and calculated by the Reed-Muench method [27]. These H3N8 viruses were named A/Mallard/Inner Mongolia/T222/2018(H3N8), A/Spot-billed duck/Inner Mongolia/T51/2016(H3N8), A/Mallard/Inner Mongolia/T75/2016(H3N8), A/baikal teal/Shanghai/SH131/2016(H3N8), A/baikal teal/Shanghai/SH-90/2013(H3N8), A/baikal teal/Shanghai/SH90-N/2016(H3N8), A/Black-winged curlew/Tianjin/CZ355/2019(H3N8), and A/Eurasian Curlew/Tianjin/CZ322/2019(H3N8), respectively, abbreviated as T222, T51, T75, SH131, SH90-O, SH90-N, CZ355, and CZ322, respectively.

### Virus genome sequences

Viral RNA was obtained using the Simply P RNA extraction kit (Bioer technology, Hangzhou, China) from allantoic fluid. The gene segments of viruses were obtained by using reverse transcription of viral RNA and subsequent PCR (primers sequences specific for these viruses available upon request). These viral gene segments were sent to Huada gene company (BGI, Beijing, China) for Sanger sequencing. Viral gene sequences were analysed by lasergene sequence analysis DNAStar software package. The wild-type genome of these strains were sequenced and uploaded into the GenBank database. The GenBank accession for the whole-genome sequences of the isolates are as follows: MT835200-MT835207 for T222, MT835216-MT835223 T51, MT835208-MT835215 for T75, MT835168-MT835175 for SH131, KJ907540.1-KJ907547.1 for SH90-O, MT835176-MT835183 for SH90-N, MT835184-MT835191 for CZ355, and MT835192-MT835199 for CZ322.

### Animals

Six-week-old females with the BALB/c genetic mice and Hartley strain female guinea pigs weighing 300–350 g were purchased from Beijing Experimental Animal Center, Beijing, China. Four-month-old female ferrets were purchased from Wuxi Cay Ferret Farm, Jiangsu, China. The ferrets used in this study were confirmed to be H3 influenza-seronegative by hemagglutination inhibition (HI) assay using chicken erythrocytes. Although the possibility of the presence of antibodies against N8 subtype influenza virus in ferrets’ serums is very low, we have not completely ruled out this possibility. All animals were acclimatized in the animal room for seven days before the experiment started and were given food and water ad libitum.

### Transmission in Guinea pigs

To investigate the transmission of wild birds-origin H3N8 influenza viruses between animals, 106 EID50 (50% egg infective doses) of each test virus in a 0.3-ml volume of medium (0.15 ml per nostril) were inoculated into groups of three guinea pigs nasally that anesthetized by ketamine (20 mg/kg) and xylazine (1 mg/kg). After 24 h, three naive animals were introduced into the same cage respectively for direct contact (abbreviated as DC) transmission and three naive animals were respectively put into the adjacent cage for airborne contact (abbreviated as AC) transmission studies. Nasal washes were collected every 2 days, beginning on day 2 d.p.i. (days post infection) and titrated in eggs. The ambient temperature is controlled at 20–22°C, the relative humidity is maintained at 40%, and the airflow was set as a speed of 0.1 m/s. Sera were collected at the end of the 3-week observation period and treated with Vibrio cholerae receptor-destroying enzyme (Denka-Seiken) before being tested for the presence of hemagglutinin inhibition (HI) antibody with 0.5% (vol/vol) chicken erythrocytes.

### Transmission in ferrets

To investigate the replication of H3N8 influenza viruses, we anesthetized two ferrets with ketamine (20 mg/kg) and xylazine (1 mg/kg) and inoculated them with 106 EID50 of T222 via intranasal route and related mutant virus in a 0.5-ml volume of medium (0.25 ml per nostril). For the respiratory droplet transmission studies, groups of three as donor animals were inoculated with 106 EID50 of test virus and housed in specially designed cages inside an isolator as described previously [28]. Twenty four hours later, three naive ferrets as exposed animals were introduced into an adjacent cage. Six ferrets were used to assess each virus: three donors and three airborne contacts. The ambient conditions for this study were set as same as in the study in guinea pigs. Nasal wash was collected from all animals every two days between days 2–10 d.p.i. or 1–9 d.p.e. (days post-exposure) to determine the virus titers. The nasal wash was also collected 24 h post-infection to see the difference in virus shedding between ferrets infected with WT and rT222-S524G mutant during early infection. Serum was separated to detect seroconversion on day 21 d.p.i. or d.p.e. by HI assay using chicken erythrocytes. Considering the stochastic process of transmission and some variations that may influence the reproduction rates, we repeated the transmission experiment using viruses generating from reverse genetics once in the ferrets.

### Phylogenetic analyses

Multiple sequence alignment was analysed using ClustalW in BioEdit version 7.2.5, and coding-region sequences were used to construct the phylogenetic trees. Phylogenetic analyses were performed using MEGA version 7.0, using the neighbor-joining method, Kimura two-parameter model. Bootstrap values were set to 1000 to evaluate the robustness of the branch support.

### Receptor-binding assays

The receptor affinity of isolated H3N8 viruses was determined by the ability to bind to chicken erythrocytes using the HA assay as described in a previous study [29]. Briefly, there are two kinds of glycans the surface membrane of chicken erythrocytes, namely the glycans on chicken erythrocytes where terminal sialic acids are attached in the α2-6 configuration (NeuAcα2, 6Gal) and the glycans on chicken erythrocytes where terminal sialic acids are attached in the α2-3 configuration (NeuAcα2, 3Gal). The chicken erythrocytes express primarily NeuAcα2, 6Gal after α2,3 sialidase enzyme treatment. Then, the HAU (hemagglutination unit) of the virus with the untreated or treated chicken erythrocytes was detected to determine whether the virus has the ability to bind NeuAcα2, 6Gal or NeuAcα2, 3Gal.

### Viruses-glycan receptor binding assay

Receptor affinity was determined using a solid-phase direct virus binding assay as previously described [30]. Briefly, influenza viruses were purified by sucrose density gradient ultracentrifugation and diluted to about 2.0 × 105 HAU/ml. Purified viruses were labelled with Alexa Fluor 555 dye (Thermo Fisher Scientific) according to the manufacturer’s instructions. The labelled virus was dialyzed in the dialysate buffer with dark agitation for 3 times, 1 h each time. The fourth dialysis was performed overnight to remove the extra Alexa Fluor 555. After dialysis, the HAU of the virus was determined again and then combined with g 80 N-glycans (Figure S1), which was printed on the N-hydroxysuccinimide-derivatized slides as previously described [31]. The slides were covered with aluminum foil and incubate in the dark at 4°C for 1 h. The slides were rinsed 3 times, the washing buffer was immediately removed, and then centrifuged briefly to dry. Data were scanned using InnoScan 1100 AL fluorescence imager (Innopsys, Carbonne, France).

### Polymerase activity assay

A dual-luciferase reporter assay system (Promega, Madison, WI, USA) was used to compare viral RNP complexes’ polymerase activities. The pCAGGS plasmids expressing wild-type PB2, PB1, PA, and NP, or mutant PB1 S524G gene with pPolI-NP plasmids expressing a reporter Firefly luciferase gene under the control of human PolI promoter and an internal control plasmid expressing Renilla luciferase were prepared. According to the manufacturer’s transfection protocol, human 293 T cells were transfected with the above plasmids using Lipofectamine® 3000 reagent (Invitrogen, L3000008). After incubation at 37°C for 24 h, the Firefly and Renilla luciferase activities of the cell lysates were measured using the Dual-Glo Luciferase Assay System (Promega). All experiments were performed in triplicate.

### Site-directed mutagenesis and virus generation

The eight segments of T222 were inserted into the bidirectional transcription vector pBD using In-Fusion® HD Cloning Kit (Takara). The Fast Mutagenesis System (TRAN) was used to create specific mutations in the PB1 gene using the following primers: forward: ATGAATCGGCTGACATGGGCATTGGAGTTACAG, reverse: CCATGTCAGCCG ATTCATTGATTCCAGATACTCCA. 293T cells were transfected with eight constructed plasmids using Lipofectamine 3000 according to the manufacturer’s instructions. Forty eight hours after transfection, the cells were disrupted, and the supernatants were collected then inoculated into 9-day-old SPF chicken embryos. After 48 h of incubation, the amnio-allantoic fluid was harvested to determine the HAU. The rescued viruses were sequenced entirely to confirm the absence of unwanted mutations.

### Pathogenicity in BALB/c mice

Eight mice were lightly anesthetized with CO_2_ and each mouse was challenged intranasally with 50 μl of virus suspension in phosphate-buffered saline (PBS) with 106 EID50 for each H3N8 avian influenza virus. Eight control mice were inoculated with 50 μl PBS. The remaining five mice were monitored for weight loss and clinical signs until 14 d.p.i.

### Virus growth in vitro

MDCK cells were infected at an MOI of 0.01 and incubated at 37°C for 1 h to compare the growth of T222 and mutant bearing S524G mutations in PB1 in vitro. Then 2 ml of complete media (DMEM) containing 2 µg/ml of TPCK trypsin and 0.15% bovine serum albumin (BSA) were added to a 6-well plate. At the indicated times post-infection, 200 μl of the supernatant was removed and replaced with an equal volume of fresh medium. The samples were stored at −70°C until viral titers were determined in MDCK cells.

### Virus growth in vivo

To compare the replication rate of rT222 and rT222-S524G mutant in vivo, we infected mice, guinea pigs, and ferrets with these viruses. Fifteen mice were anesthetized with CO_2_ and inoculated intranasally with 50 µl of PBS containing 106 EID50 rT222 viruses. Lungs, trachea, and nasal turbinate were extracted from three euthanized mice at 24, 48, 72, 96, and 120 h post-infection. Three guinea pigs were inoculated intranasally with 300 µl of PBS containing 106 EID50 rT222 viruses, lungs, trachea, and nasal turbinate were collected at 24 h post-infection. Five ferrets were inoculated intranasally with 500 µl of PBS containing 106 EID50 rT222 viruses. Lung, trachea, rostral respiratory epithelium, caudal respiratory epithelium, olfactory epithelium, and tonsil were collected 24 h post-infection. All collected tissue was homogenized in 0.1% w/v gelatin-PBS and preserved at – 80°C until viral titers determination. Another two ferrets were euthanized on day 4 p.i. Then, lungs, tracheas, and nasal turbinates were sampled and fixed in 10% neutral buffered formalin, embedded in paraffin, and sectioned. Sections were stained using hematoxylin and eosin for histology. The above animal experiments were performed in parallel with the rT222-S524G mutant.

### Statistical analysis

For comparisons of two groups with a single time point, an unpaired *t*-test followed with a Welch’s correction for unequal standard deviations was used. A two-way analysis of variance (ANOVA) followed by a Sidak’s post-test to adjust for multiple comparisons was performed for comparisons of two groups with multiple time points. All statistical analyses were performed in GraphPad Prism 7.04. The asterisks shown in the figures refer to the level of significance (zeros after the decimal point of the *p*-value): *p ≤ 0.05; **p ≤ 0.01; ***p ≤ 0.001; ****p ≤ 0.0001. No samples or animals were excluded from the analysis.

## Results

### Genetic analyses of wild bird-origin H3N8 IAVs

To investigate the genetic relationship of the viruses from our surveillance from 2013 to 2019 along East Asian-Australasian migratory bird flyway, two H3N8 viruses from a shorebird of the Charadriiformes order and six H3N8 isolates from a bird of the Anseriformes order were sequenced, and phylogenetic analysis was performed.

**Figure 1.**
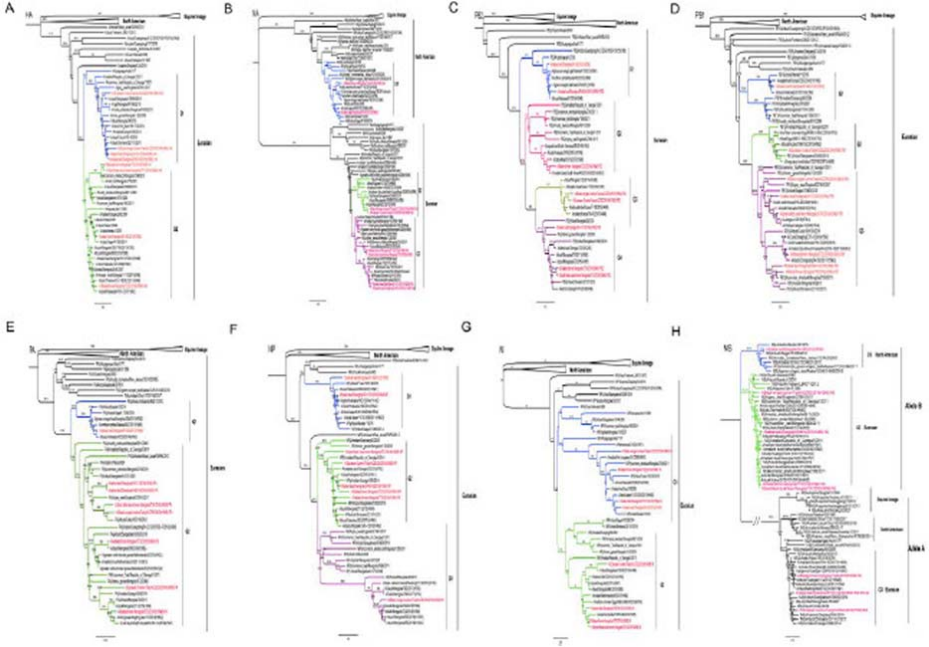
Phylogenetic analyses of wild bird-origin H3N8 influenza A viruses. The phylogenetic tree for each gene segment was inferred using a maximum likelihood method using a Gamma model of rate heterogeneity and a GTR substitution model. The taxa name of viruses sequenced in this study is shown with red in the phylogenetic tree.

As shown in Figure 1, the HA genes of the eight H3N8 avian viruses are markedly distinct from classical equine influenza virus lineage and North American avian influenza virus lineage. The HA genes of the eight viruses share 90.6%-99.3% identity at the nucleotide level and formed 2 different groups in the phylogenetic tree (95% sequence identity cutoffs were used to categorize each gene group in all of the phylogenetic trees). The NA genes of the eight avian viruses share 77.7%–99.9% identity at the nucleotide level and formed three groups in the phylogenetic tree. The six internal genes of the H3N8 viruses show distinct diversity, with the basic polymerase 2 (PB2), basic polymerase 1 (PB1), acidic polymerase (PA), nucleoprotein (NP), matrix (M), and nonstructural protein (NS) genes of the eight viruses sharing 91.3%–99.7%, 91.9%–98.2%, 93.0%–100%, 90.0%–98.9%, 95.1%–99.9%, and 72.0%–99.5% identity, respectively, at the nucleotide level. The PB2, PB1, PA, NP, M, and NS genes formed 4, 3, 2, 3, 2, and 3 groups in the phylogenetic tree. Based on genomic diversity, we categorized the eight viruses into seven different genotypes (Table S1). The largest differences were observed in the NA and NS phylogenies, where the NA segments of the T222 and SH90-O viruses and NS segment of SH90-O clustered together with the American lineage, unlike the other gene segments which clustered with Eurasian lineage (Figure 1).

Several putative mammalian adaptive mutations occurred in these H3N8 viruses (Table S2). We identified I155T substitution in HA [32] that increases affinity for the human-type receptor in all 8 strains. T159N in HA, a hallmark that increases the replication or virulence of avian influenza viruses [33], occurs in isolates T71, T75 and SH90-O. In PB1, the amino acid 207 K, 269S, 436Y, 473 V and 677 T were conserved in 8 strains. T222 was equipped with an additional mutation S216G, which did not occur in all 8 isolated viruses. Another mutation R584H appeared in SH90-N. In PA, adaptive mutations such as L295P, N383D, M423I, V476A, A515 T, V630E [34–37] were detected in all isolates. Mutation V100A appeared in the only CZ355 in PA. CZ322 had another mutation E382D in PA. None of the substitutions for mammalian adaptation in PB2 such as G590S, Q591R, E627 K or D701N [38] were found. Residue 30D and 215A in M1 [39] segment were conserved in all strains. In NS1, the 42S were detected in 8 strains and the NS1-213S which endows airborne transmission [25] did not appear in all the 8 isolates.

### The H3N8 AIVs bind to both avian-type and human-type receptors

A species barrier for the influenza A virus is the differential receptor binding specificity. The switch of receptor binding specificity from a2,3-linked to a2,6-linked is a critical determinant for avian influenza gain the cross-species transmission to human. Firstly, the receptor-binding specificities of the eight viruses were determined by HA assays. With chicken red blood cells treated with or without α2,3 sialidase, similar in HA titers of T222, T75, SH131, SH90-N, CZ355, CZ322 proved that these viruses bind to avian-type and human-type receptors (Figure S1). The loss of agglutination activities of the T51 and SH90-O virus with chicken red blood cells treated with α2,3 sialidase indicated that the two viruses could not bind human-type receptors (Figure S1).

**Figure 2.**
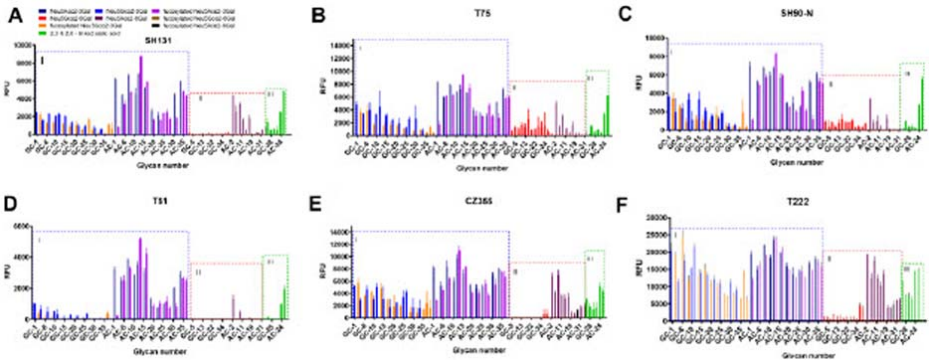
Glycan array analysis of wild bird-origin H3N8 influenza viruses: (A) SH131, (B) T75, (C) SH90-N, (D) T51, (E) CZ355, and (F) T222. The frames I to III represent different glycans categories: I, alpha-2,3-linked sialic acid glycans; II, alpha-2,6-linked sialic acid glycans; III, alpha-2,3- and alpha-2,6-linked sialic acid glycans. Glycans on the microarray are grouped according to SA linkage and glycans modification: Neu5Gcα2-3Gal (blue), Neu5Acα2-3Gal (Navy blue), Neu5Gcα2-6Gal (Red), Neu5Acα2-6Gal (Dark Brown), fucosylated Neu5Acα2-3Gal (Purple), fucosylated Neu5Gcα2-3Gal (Orange), fucosylated Neu5Acα2-6Gal (Black), fucosylated Neu5Gcα2-6Gal (Brown), and 2,3 & 2,6-linked sialic acid (Green). Vertical bars denote the fluorescence binding signal intensity on the array. The structures of each numbered glycan are found in Figure S5.

In order to further analyse the receptor specificity of H3N8 avian influenza virus used in this study, we performed N-glycan array assay comprised of representative common IAV binding determinants containing either NeuAc or NeuGc glycans. We found that the H3N8 avian influenza viruses T222, T75, SH131, SH90-N, and CZ355 strain can bind to NeuAcα2, 6Gal and NeuAcα2, 3Gal, respectively (Figure 2). The H3N8 avian influenza virus T51 strain can bind to NeuAcα2, 3Gal with low binding activity, but it was difficult to bind to NeuAcα2, 6Gal. SH131 and CZ355 strains had binding affinity for α2,6-linked Neu5Acα, while SH90-N, T75, and T222 strains had binding affinity for α2,6-linked Neu5Acα and α2,6-linked Neu5Gcα. T222 strain had the strongest binding ability to various N-glycans. In terms of binding strength, the binding affinity of T222 strain with α2,3-linked Neu5Acα was stronger than that of Neu5Gcα and α2,6-linked Neu5Acα (Figure 2). These findings indicated that some H3N8 strains used in this study can bind to α2,6-linked sialic acid glycans receptors. Taken together, these results suggest that the H3N8 viruses isolated from wild birds may have the ability to spread among mammals.

### Transmissibility of wild bird-origin H3N8 viruses in Guinea pigs

**Figure 3.**
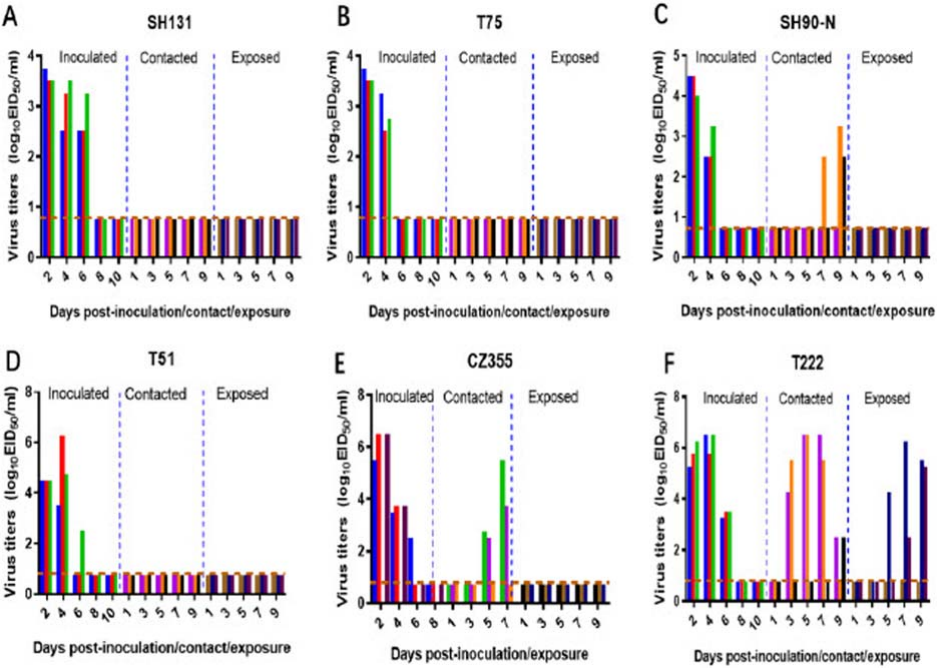
Transmission of H3N8 avian influenza viruses in guinea pigs. Viral titers in the nasal washes of guinea pigs after infection with avian H3N8 influenza A viruses. Groups of three guinea pigs were inoculated with 10^6^ EID_50_ of test virus in a 300-μl volume (150-μl per nostril). 24 h later, three naive guinea pigs were introduced into the same cage to test for direct-contact transmission, and another three naive guinea pigs were put into the adjacent cage for respiratory droplet transmission studies. Nasal washes were collected at the indicated times for detection of virus shedding. Each color bar represents the virus titers from an individual animal. Dashed lines represent the limit of detection.

To investigate the mammalian transmission ability of H3N8 avian influenza virus, we evaluated the transmissibility of T222, T75, SH131, SH90-N, CZ355, and T51 viruses among guinea pigs. As shown in Figure 3, the virus was detected in the nasal washes of all guinea pigs inoculated with H3N8 virus on 2 and 4 d.p.i. The SH90-N and CZ355 viruses transmitted among guinea pigs through direct contact (Figure 3(C–E)). Two of the three DC guinea pigs shed high titers of CZ355 viruses on days 5 and 7 p.c. The virus was recovered from the nasal washes of one of the three guinea pigs that were contacted to the SH90-N-inoculated guinea pigs on 7 d.p.c. and two of the three DC guinea pigs were positive on 9 d.p.c. Of special note, the T222 virus transmitted very rapidly to three DC guinea pigs, as viral titers were detected in that guinea pigs as early as 3 d.p.c. (Figure 3(F)). Most importantly, the virus was also detected in the nasal washes of two of three AC guinea pigs exposed to T222-inoculated guinea pigs between days 5 and 9 d.p.e., which means T222 has efficient respiratory droplet transmission between guinea pigs. Specific antibody against H3N8 was detected in all the virus-inoculated animals and in all contacted and exposed animals that were virus-positive (Figure S3). These results indicate that three of the six H3N8 viruses tested can transmit between guinea pigs through direct contact, and one of them transmitted efficiently via respiratory droplets.

### Amino acid substitutions detected between T222 and T222-G1

To determine putative airborne transmission markers for T222 isolate, we sequenced the virus that were recovered from DC and AC guinea pigs. The S524G mutation of PB1 was detected in the samples that were collected from DC and AC guinea pigs at 3 d.p.c. and 5 d.p.e. (Table 1). We found no other amino acid changes in viruses isolated from DC or AC or T222-inoculated guinea pigs. T222 and T222-G1 with the S524G mutation in PB1 were used for airborne transmission studies in ferrets.

**Table 1.** Mutations of H3N8 viruses isolated in the nasal washes of guinea pig in the transmission study.

The nasal washes of guinea pig	Amino acid change(s) detected at the indicated time						
	Day 2 p.i.	Day 4 p.i.	Day 6 p.i.	Day 3 p.e.	Day 5 p.e.	Day 7 p.e.	Day 9 p.e.
T222-I1	*	*	PB1 S524G				
T222-I2	*	PB1 S524G	PB1 S524G				
T222-I3	*	PB1 S524G	PB1 S524G				
T222-C1				PB1 S524G	PB1 S524G	PB1 S524G	PB1 S524G
T222-C2				PB1 S524G	PB1 S524G	PB1 S524G	/
T222-C3				/	/	/	PB1 S524G
T222-E1				/	/	/	/
T222-E2				/	PB1 S524G	PB1 S524G	PB1 S524G
T222-E3				/	/	PB1 S524G	PB1 S524G

* No mutation detected.

/ No virus isolated.

### Airborne transmission of T222 and T222-G1 in ferrets

**Figure 4.**
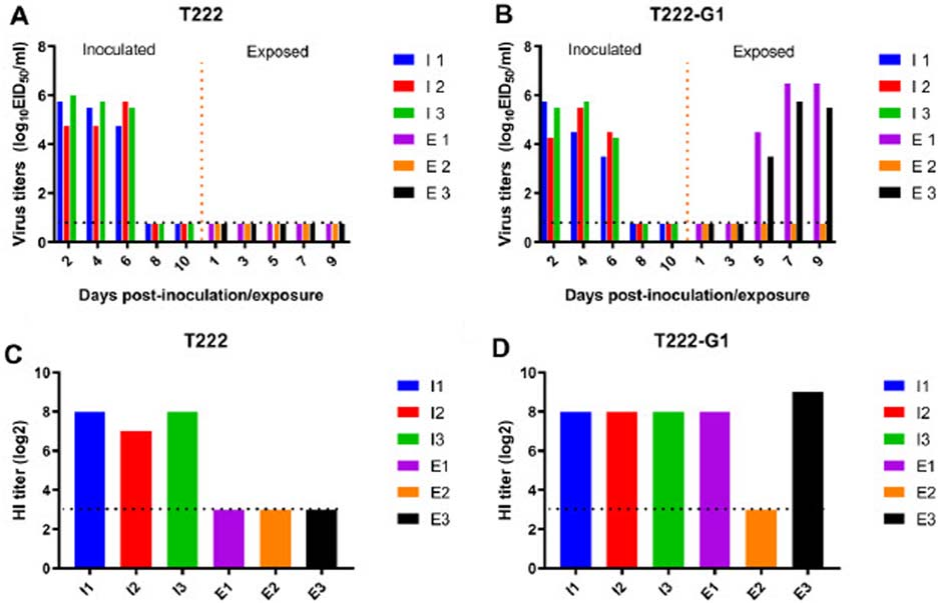
Airborne transmission of T222 (A) and T222-G1 (B) in ferrets. Ferrets (*n* = 3 per group) were inoculated with 10^6^ EID_50_ of test virus in a 500-μl volume (250 μl per nostril). Twenty-four hours later, three naive ferrets were put into the adjacent cage for respiratory droplet transmission studies. After infection with the test virus, the nasal washes of ferrets were collected at the indicated d.p.i. for viral titers (A and B). HI antibody titers of the animals are shown (C and D). Each column represents a single ferret on the indicated d.p.i. Dashed lines represent the limit of detection.

Transtablemission of a virus between ferrets via the airborne route is key for the predicting potential transmission in humans. To determine the role of S524G mutations of PB1 in airborne transmissibility between mammals, we assessed the transmissibility of T222 and T222-G1 in ferrets through the airborne route by separating animals in an adjacent cage. As shown in Figure 4, T222 did not transmit via the airborne route between ferrets, while T222-G1 showed airborne transmission with high virus titers in both inoculated and two of three AC ferrets. Ferrets inoculated with the T222 virus were observed increasing temperature and sneezing from 2 to 4 d.p.i. Ferrets inoculated with the T222-G1 mutant virus were observed increasing temperature and sneezing from 2 to 4 d.p.i. Two of three AC ferret were observed sneezing on day 6 d.p.i. and increasing temperature from 8 to 10 d.p.i. (Figure S3(A, B)). All of the inoculated and the exposed ferrets that shed viruses seroconverted on day 21 d.p.i. or d.p.e. (Figure 4(C, D)). These results indicate that the T222-G1 virus acquired the airborne transmissibility between ferrets, with which T222 was not equipped.

To further determine whether any key amino acid changes that enhanced the transmission ability during replication of T222 and T222-G1 in ferrets, we performed deep sequencing of nasal wash recovered from inoculated and AC ferrets. The Q226L and N188D mutations of HA were respectively detected in the samples collected on day 4 d.p.i. from three T222 virus inoculated ferrets (Table S4). T159N and Q226L mutation were introduced into the virus that recovered from three inoculated ferrets on 4 d.p.i. and two of three exposed ferrets on 5 d.p.e. (Table S4). Residues 159, 188 and 226 are important to virus receptor binding specificity and, therefore, may influence their transmissibility in mammals [33, 40, 41]. Considering that Q226L appeared in ferrets challenged by T222 and T222-G1, the S524G mutation in PB1 should be the key molecular mechanism leading to the different transmission results.

### The S524G mutation in PB1 increases the airborne transmissibility of H3N8 viruses in ferrets

**Figure 5.**
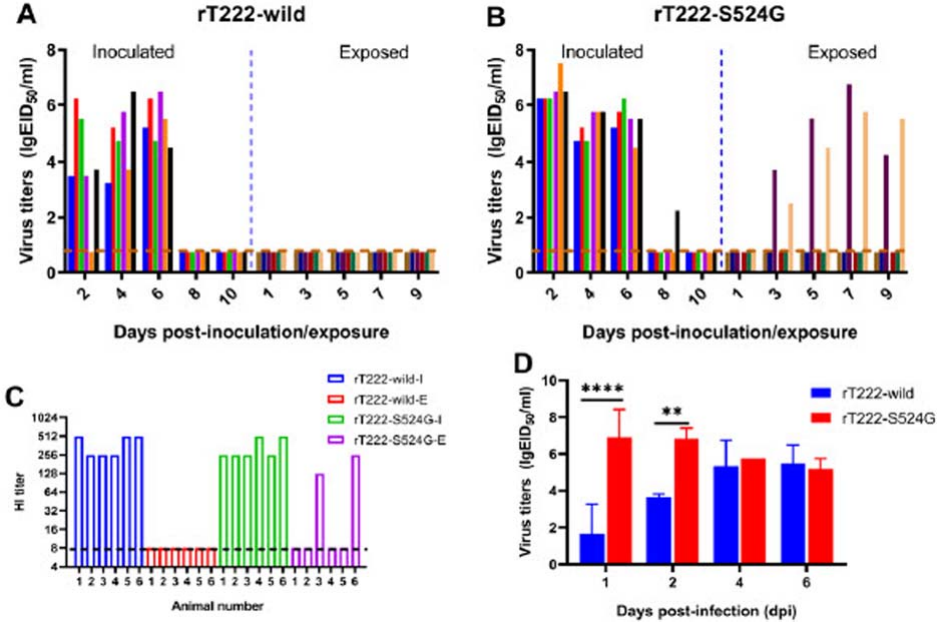
Airborne transmission of rT222 and rT222-S524G rescued by reverse genetics in ferrets. The vertical axis represents titers of influenza viruses recovered from nasal washes of the virus-inoculated and exposed ferrets (A and B). HI antibody titers of the animals are shown C. Each column represents individual animals. Dashed lines represent the limit of detection. Average virus titers in the nasal wash of ferret inoculated with the virus at the indicated time are shown D.

To study the effect of a PB1- S524G mutation in airborne transmission, we used reverse genetics to rescue an rT222-wild virus and virus-containing PB1-S524G (rT222-S524G). The airborne transmissibility of these viruses was then tested in ferrets. Similar to the wild-type T222, the virus was isolated, and seroconversion was detected in all the ferrets infected with the rT222-wild virus but not in exposed ferrets (Figure 5(A)). In the rT222-S524G group, the virus was isolated from all of the infected and two of six exposed animals (Figure 5(B)). Seroconversion occurred in all inoculated animals and in two of six exposed ferrets (Figure 5(C)), which was consistent with virus shedding. Besides, infectious virus titers in nasal washes from ferrets inoculated with rT222-S524G viruses were significantly higher than rT222-wild viruses at 1and 2 d.p.i. (*p* < 0.0001 at 1 d.p.i. and *p* < 0.01 at 2 p.d.i., respectively) (Figure 5(D)). These results indicate that the S524G mutation in PB1 conferred the airborne transmissibility of H3N8 viruses in ferrets, which may be attributed to the higher infectious viral titers in nasal washes in early infection.

### The S524G mutation in PB1 enhances polymerase activity and increase viral replication in mammalian cells

**Figure 6.**
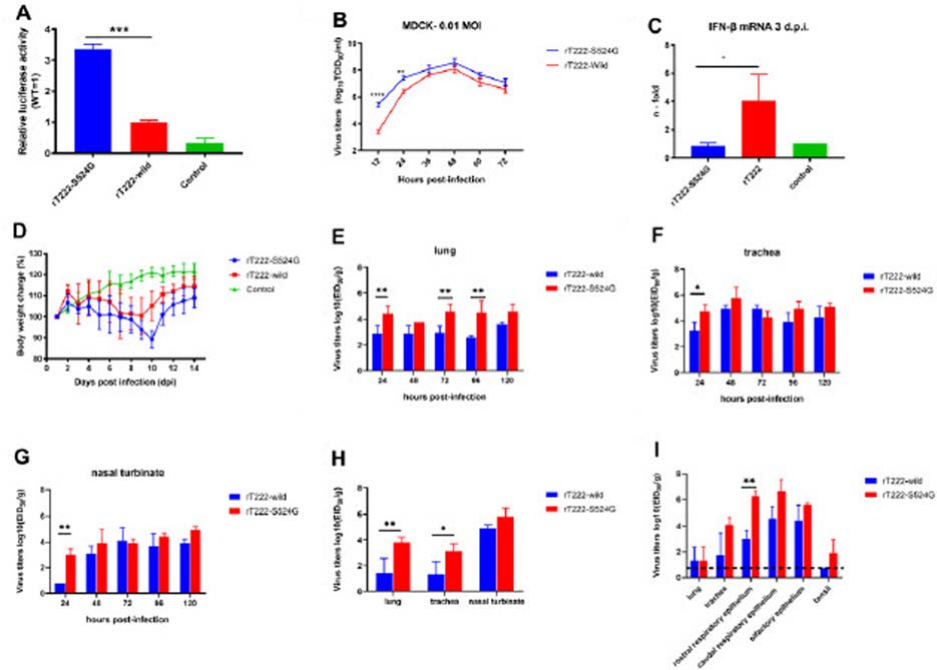
The replication of rT222 and mutant rT222-S524G *in vitro* and *in vivo*. (A) Polymerase activity was measured in 293T cells at 37°C. Cells not expressing PB1 served as control. RNP polymerase activities of wild-type and mutant viruses in 293T cells, transfected with polymerase plasmids (PB1/PB1-S524G, PA, NP, and PB2), were determined by minigenome replication assays. (B) Replication kinetics of rT222-wild and rT222-S425G in MDCK cells. Cells were infected with viruses at MOI of 0.01. A significant difference was found between infectivity titers for rT222-wild and rT222-S425G in MDCK cells in 12 and 24 hours post-infection. (C) Bodyweight change differences between mice inoculated with rT222-wild and rT222-S425G virus. (D) The IFN-β expression in the lung of inoculated mice with rT222 and rT222-S524G. The mean virus titers in the lung, trachea, and nasal turbinate tissues of infected mice at the indicated time are shown in (E), (F), and (G), respectively. The mean virus titers in the respiratory organs in guinea pigs and ferrets at 24 h post-infection are shown in (H) and (I), respectively.

Avian viruses must overcome the poor polymerase activity to infect humans. As a component of the ribonucleoprotein complex, PB1 protein plays an important role in polymerase activity. Viral minigenome polymerase assays were performed to identify whether PB1-S524G mutation enhances the RNP polymerase activity. As shown in Figure 6(A), the PB1 mutation S524G enhances polymerase activity 3.3-fold in human cells at 37°C (*p* < 0.01). The result indicates that PB1-S524G is an adaptive mutation that enhances the polymerase activity of the H3N8 avian influenza virus in human cells. To evaluate the effect of S524G mutation in PB1 on the growth properties of virus, we compared the growth kinetics on MDCK cells. Growth kinetics of wild-type (rT222) and mutant (rT222-S524G) H3N8 viruses were determined in MDCK cells at an m.o.i. (multiplicity of infection) of 0.01 over 72 h. rT222-S524G was more productive by about a hundred-fold and ten-fold relative to rT222 at 12 and 24 h, respectively (Figure 6(B)). The result indicated that the mutation S524G significantly enhanced the growth ability of the H3N8 virus in MDCK cells at early infection.

### The S524G mutation in PB1 altered viral replication in the respiratory tract in mammals

**Figure 7.**
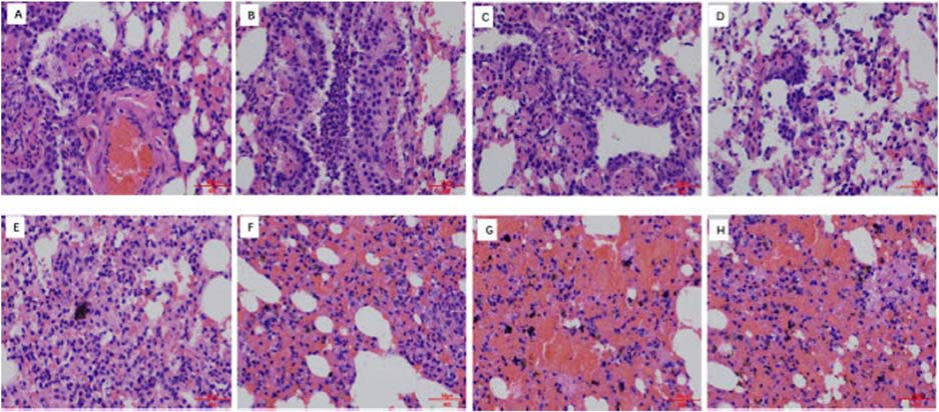
Histological lesions caused by H3N8 viruses in the lungs of ferrets. Ferrets were euthanized on 4 d.p.i. with 10^6^ EID_50_ of test virus, and the lungs were collected for pathological study. The lungs of rT222 virus-inoculated animals showed only mild histopathological changes (H&E staining) (A-D), whereas the lungs of rT222-S524G(E-H), virus-inoculated ferrets showed severe pathological lesions (H&E staining). Images were taken at a 40×magnification.

To test whether the phenotypes of rT222 and rT222-S524G seen in vitro are also manifested in vivo, we evaluated the titers of these viruses in different organs in mice, guinea pigs and ferrets. During the 14 days of follow-up, mice infected with rT222-wild did not experience bodyweight loss while mice inoculated with rT222-S524G virus showed some weight loss of around 10.79% with no mortality (Figure 6(D)), which was consistent with the results of the RNP polymerase activity and growth kinetics assays. As shown in Figure 6(E–G), rT222-S524G viruses exhibited enhanced replication in the lung, trachea, and nasal turbinate tissues of infected mice at 24 h post-infection (*p* < 0.01). At 72 and 96 h post-infection, the virus titers in the lungs of mice inoculated with rT222-S524G viruses have a higher level than those of mice infected with rT222-wild viruses (Figure 6(E)). In the guinea pigs model, 24 h post-infection, the virus titers in the lung, and trachea tissue were significantly higher infected with rT222-S524G than T222-wild while there is no statistical difference in nasal turbinate (Figure 6(H)). There were significantly different virus titers in rostral respiratory epithelium between ferrets inoculated with different viruses at 24 h post-infection (*p* < 0.01) (Figure 6(I)). The S524G mutation in PB1 attenuates virus induced IFN-β expression in mice (*p* < 0.05) (Figure 6(C)). Consistent with results in mice, compared with ferrets inoculated with T222-wild viruses, PB1-S524G mutant caused more severe pathological lesions in the lungs of ferrets (Figure 7). Collectively, PB1-S524G mutation in the H3N8 virus causes more severe infection in mammals. These results confirm that the PB1-S524G adaptation optimizes the in vivo replication ability of H3N8 viruses in mammalian host species, especially in early infection.

## Discussion

The mammalian pathogenicity and transmission ability of avian influenza viruses isolated from poultry have been evaluated in a large number of previous studies [42–45]. However, the transmission ability of viruses isolated from wild birds in mammals is poorly understood. A previous study has confirmed that some H1N1 avian influenza viruses isolated from the Charadriiformes in contrast to those of Anseriformes order can transmit via air between mammals [46], strongly indicating that avian influenza viruses with mammal-to-mammal transmission ability have existed in wild birds. Furtherly, Koçer et al. found that NS residue 213S is important for viruses from Charadriiformes order to obtain airborne transmissibility in mammals [25].

Influenza virus transmission in wild and domestic animals and humans is intimately connected. A shift host from aquatic birds to landfowl or mammals is an important step for AIVs to obtain the potential for subsequent pandemic spread. H3N8 AIVs transfer into new hosts, having caused outbreaks in equine and canine and sporadic infections in pigs, camels, and harbour seals. AIVs may be transmitted from animals to humans through an intermediate host. The intermediate host allows influenza A viruses from different species to mix and create a new virus by antigenic shift. Zhu et al. found that horses could be coinfected by both avian and equine H3N8 viruses, making genetic reassortment possible [47]. The selection pressures of hosts promote virus acquire adaptive changes during long-term circulation of avian-origin IAVs in mammals. The adaptation in gene segments contributes to virus replication and transmission in mammals.

Given the wide host range and the potential acquisition of mammalian adaptation phenotypes, which have a significant impact on public health, the H3N8 avian influenza virus, as one of the most commonly found subtypes in wild birds need to be surveyed and analysed. Here, we characterized two H3N8 viruses from a shorebird of the Charadriiformes order and six H3N8 isolates from a bird of the Anseriformes order. Phylogenetic analyses suggested that the H3N8 viruses have undergone frequent reassortment and formed complicated genotypes. Of note, the NA segments of T222 and SH90-O viruses and the NS segment of SH90-O clustered together with American lineage, unlike the other gene segments which clustered within the Eurasian lineage. Intercontinental viral dispersal induced by migratory birds has been reported in other [48, 49]. The intercontinental gene segment exchange role in the evolution and transmission of the H3N8 avian influenza virus needs further study.

A shift in HA from avian- to human-type receptor specificity is a prerequisite for avian viruses to replicate and be efficiently transmitted in humans [50, 51]. In this study, six of eight H3N8 viruses isolated naturally from wild birds have acquired the ability to bind to the human-type receptor, although the affinity for α-2,6-linked SAs was lower than that for α-2,3-linked SAs, highlighting the potential of H3N8 influenza viruses for avian-to-human transmission. Previous studies by the Robert P. de Vries’ research groups identified N-glycolylneuraminic acid as a receptor of influenza A viruses, and NeuGc specificity represents a species barrier [52]. Microarray analysis further confirmed that the T222 virus representing the G1 genotype binds to N-glycolyl, and N-acetyl, which agrees with the repeated interspecies transmission of H3N8 influenza viruses from birds to mammals. In addition to the type of glycosidic linkage, the type of sialic acid (Neu5Gc or 5-N-acetyl neuraminic acid) also plays an important role in host tropism of influenza A viruses [30]. Of note, a study suggested that mutation W222L and S227R seem to affect IAV binding to Neu5Gcα2,3Gal with the fucosylated motif [53]. Amino acid sequence comparison of the H3N8 HA showed all the viruses tested in this study did not contain the above mutations (Table S3). The motif of N-glycan of H3N8 avian influenza virus preferential binding to and the molecular determinants still needs to be studied further.

Guinea pigs and ferrets have been widely used to research the genetic and phenotypic traits required for AIV to transmit in a mammalian host. Three of eight viruses exhibited efficient direct contact transmission without prior adaptation in guinea pigs in our study. One virus transmitted efficiently via respiratory droplets in guinea pigs without showing any airborne transmission in ferrets. The difference in receptor distribution in the guinea pigs respiratory tract and ferret may explain this difference. The different experimental results suggest that it is necessary to characterize AIVs’ pandemic potential using multiple animal models.

In this study, we found that the S524G mutation of PB1 was detected in the samples collected from DC and AC animals, and the virus recovered from contact guinea pigs can transmit efficiently in ferrets via respiratory droplets while the parent virus T222 cannot.

The growth of rT222 and rT222-S524G in vivo confirm that the PB1-S524G increased replication of virus with varying efficiency in early infection in the respiratory tract of different animal models with. The transmission experiments in ferret proved that S524G mutation of PB1 was vital for airborne transmission. The efficiency of aerosol transmission was improved by PB1 S524G mutation in ferrets but not in guinea pigs (Figure S4), which was reasoned the higher growth of rT222-S524G in the upper respiratory of ferrets than wild virus rT222.

Furthermore, Q226L mutation was detected both in ferrets inoculated with T222 (non–airborne-transmissible viruses) and T222-G1 (airborne-transmissible viruses). Several studies have shown that some viruses gained Q226L/G228S mutations in HA protein during replication resulting in enhanced binding to α-2,6 sialic acid receptors and thus acquire mammalian transmission [24, 54]. This study also determines that T222-G1 with Q226L exhibits enhanced affinity for human-type sialic acid receptors than T222 (data available upon request). Therefore, only the Q226L mutation cannot allow the H3N8 avian influenza virus to be airborne transmission among ferrets. The T222 virus quickly acquired the Q226L mutation after infecting ferrets, so whether just S524G in PB1 confers airborne transmissibility could not be determined. Given the determinants of airborne phenotype for influenza virus is a polygenic trait, PB1 G524 alone may be insufficient for airborne transmissibility. PB1 S524G combined with Q226L occurred during infection, which enhances affinity to α2,6 receptors cooperatively contribute to airborne transmission of H3 viruses in ferrets.

In summary, we have revealed the potential transmission in mammals via an airborne route of H3N8 viruses from a wild bird reservoir. We have also demonstrated a role for PB1 in facilitating the airborne transmission in these viruses and characterized the impaction of mutations in PB1 in viral replication and pathogenicity. Our results emphasize the importance of research on how readily the avian viruses in wild birds can gain genetic markers of transmissibility in mammals and inform us that continuous surveillance of the enzootic dynamics of virus circulating in wild bird reservoirs is warranted.

## Supplementary Material

Table_S4.docx

Table_S3.docx

Table_S2.docx

Table_S1.docx

Figure_S5.docx

Figure_S4.docx

Figure_S3.docx

Figure_S2.docx

Figure_S1.docx
