# Supplementary material for: PB1 S524G mutation of wild bird-origin H3N8 influenza A virus enhances virulence and fitness for transmission in mammals
Source: Emerg Microbes Infect. 2021 Jun 6;10(1):1038–51. doi: 10.1080/22221751.2021.1912644 (PMC8183522; doi:10.1080/22221751.2021.1912644)
Supplement: Table_S4.docx [file TEMI_A_1912644_SM6440.docx]

**Table S4.** Mutations of H3N8 viruses isolated in the nasal washes of ferrets in the transmission study.

| Virus | Ferret | Amino acid change(s) detected in HA at the indicated times | | | | | |
| --- | --- | --- | --- | --- | --- | --- | --- |
|  |  | Day 2 p.i. | Day 4 p.i. | Day 6 p.i. | Day 3 p.e. | Day 5 p.e. | Day 7 p.e. |
|  | Inoculated #1 | * | † | † |  |  |  |
|  | Inoculated #2 | * | † | † |  |  |  |
|  | Inoculated #3 | * | Q226Q/L,  N188D | Q226Q/L,  N188D |  |  |  |
| T222 | Exposed #1 |  |  |  | / | / | / |
|  | Exposed #2 |  |  |  | / | / | / |
|  | Exposed #3 |  |  |  | / | / | / |
|  | Inoculated #1 | † | † | † |  |  |  |
|  | Inoculated #2 | † | † | † |  |  |  |
|  | Inoculated #3 | * | T159N,  Q226L | T159T/N,  Q226L |  |  |  |
| T222-G1 | Exposed #1 |  |  |  | † | † | † |
|  | Exposed #2 |  |  |  | / | / | / |
|  | Exposed #3 |  |  |  | T159N,  Q226L | T159N,  Q226L | T159N,  Q226L |

* No mutation detected;

/ No virus isolated;

† Not detected.
