# Supplementary material for: PB1 S524G mutation of wild bird-origin H3N8 influenza A virus enhances virulence and fitness for transmission in mammals
Source: Emerg Microbes Infect. 2021 Jun 6;10(1):1038–51. doi: 10.1080/22221751.2021.1912644 (PMC8183522; doi:10.1080/22221751.2021.1912644)
Supplement: Table_S3.docx [file TEMI_A_1912644_SM6439.docx]

**Table S3.** Amino acid residue difference in HA may explain variation in receptor binding specificity of wild bird-origin H3N8 influenza virus.

| Virus | HA amino acid residues（ H3 numbering） | | | | | | | | | | | | | | | | | |
| --- | --- | --- | --- | --- | --- | --- | --- | --- | --- | --- | --- | --- | --- | --- | --- | --- | --- | --- |
|  | 130 | 135 | 137 | 147 | 157 | 158 | 159 | 184 | 188 | 189 | 190 | 192 | 193 | 222 | 225 | 226 | 227 | 228 |
| T222 | V | G | N | F | S | G | T | H | N | Q | E | T | N | W | G | Q | S | G |
| T51 | V | G | N | F | S | G | N | H | N | Q | E | T | N | W | G | Q | S | G |
| T75 | V | G | N | F | S | G | N | H | N | Q | E | T | N | W | G | Q | S | G |
| SH131 | V | G | G | F | S | G | S | H | N | Q | E | T | N | W | G | Q | S | G |
| SH90-O | V | G | N | F | S | G | N | H | N | Q | E | T | N | W | G | Q | S | G |
| SH90-N | V | G | G | F | S | G | S | H | N | Q | E | T | N | W | G | Q | S | G |
| CZ355 | V | G | G | F | S | G | S | H | N | Q | E | T | N | W | G | Q | S | G |
| CZ322 | V | G | G | F | S | G | S | H | N | Q | E | T | N | W | G | Q | S | G |
