# Supplementary material for: PB1 S524G mutation of wild bird-origin H3N8 influenza A virus enhances virulence and fitness for transmission in mammals
Source: Emerg Microbes Infect. 2021 Jun 6;10(1):1038–51. doi: 10.1080/22221751.2021.1912644 (PMC8183522; doi:10.1080/22221751.2021.1912644)
Supplement: Table_S2.docx [file TEMI_A_1912644_SM6438.docx]

**Table S2.** The host adaptive mutations in the wild bird-origin H3N8 influenza virus.

| **Protein** | **Residue** | **Adaptive**  **mutation** | **T222** | **T51** | **T75** | **SH131** | **SH90-O** | **SH90-N** | **CZ355** | **CZ322** |
| --- | --- | --- | --- | --- | --- | --- | --- | --- | --- | --- |
| PB1 | 207 | K207R[1] | K | K | K | K | K | K | K | K |
|  | 216 | S216G[2] | G | S | S | S | N | S | S | S |
|  | 436 | Y436H[1] | Y | Y | Y | Y | Y | Y | Y | Y |
|  | 473 | L473V[3] | V | V | V | V | V | V | V | V |
|  | 584 | R584Q[4] | R | R | R | R | R | H | R | R |
|  | 621 | Q621R[5] | Q | Q | Q | Q | Q | Q | Q | Q |
| PA | 28 | P28L[6] | P | P | P | P | P | P | P | P |
|  | 100 | V100A[7] | V | V | V | V | V | V | A | V |
|  | 277 | S277H[8] | F | S | S | S | S | S | S | S |
|  | 295 | L295P[9] | P | P | P | P | P | P | P | P |
|  | 382 | E382D[10] | E | E | E | E | E | E | E | D |
|  | 383 | N383D[11] | D | D | D | D | D | D | D | D |
|  | 400 | P400L[12] | P | S | S | S | S | S | S | S |
|  | 423 | M423I[12] | I | I | I | I | I | I | I | I |
|  | 476 | V476A[13] | A | A | A | A | A | A | A | A |
|  | 630 | V630E[13] | E | E | E | E | E | E | E | E |
| M1 | 30 | N30D[14] | D | D | D | D | D | D | D | D |
|  | 215 | T215A[14] | A | A | A | A | A | A | A | A |
| M2 | 31 | S31N/D[15] | S | S | S | S | S | S | S | S |
| NS1 | 42 | P42S[16] | S | A | A | S | A | A | A | S |

Reference

1. Hulse-Post D, Franks J, Boyd K, et al. Molecular changes in the polymerase genes (PA and PB1) associated with high pathogenicity of H5N1 influenza virus in mallard ducks. Journal of virology. 2007;81(16):8515-8524.

2. Lin R-W, Chen G-W, Sung H-H, et al. Naturally occurring mutations in PB1 affect influenza A virus replication fidelity, virulence, and adaptability. Journal of biomedical science. 2019;26(1):1-14.

3. Xu C, Hu W-B, Xu K, et al. Amino acids 473V and 598P of PB1 from an avian-origin influenza A virus contribute to polymerase activity, especially in mammalian cells. Journal of general virology. 2012;93(3):531-540.

4. Tamuri AU, Dos Reis M, Hay AJ, et al. Identifying changes in selective constraints: host shifts in influenza. PLoS Comput Biol. 2009;5(11):e1000564.

5. Mostafa A, Kanrai P, Ziebuhr J, et al. The PB1 segment of an influenza A virus H1N1 2009pdm isolate enhances the replication efficiency of specific influenza vaccine strains in cell culture and embryonated eggs. Journal of General Virology. 2016;97(3):620-631.

6. Chen G-W, Chang S-C, Mok C-K, et al. Genomic signatures of human versus avian influenza A viruses. Emerging infectious diseases. 2006;12(9):1353.

7. Naffakh N, Massin P, Escriou N, et al. Genetic analysis of the compatibility between polymerase proteins from human and avian strains of influenza A viruses. Microbiology. 2000;81(5):1283-1291.

8. Hu Y-J, Tu P-C, Lin C-S, et al. Identification and chronological analysis of genomic signatures in influenza A viruses. PloS one. 2014;9(1):e84638.

9. Ducatez MF, Ilyushina NA, Fabrizio TP, et al. Both influenza hemagglutinin and polymerase acidic genes are important for delayed pandemic 2009 H1N1 virus clearance in the ferret model. Virology. 2012;432(2):389-393.

10. Taubenberger JK, Reid AH, Lourens RM, et al. Characterization of the 1918 influenza virus polymerase genes. Nature. 2005;437(7060):889-893.

11. Song J, Xu J, Shi J, et al. Synergistic effect of S224P and N383D substitutions in the PA of H5N1 avian influenza virus contributes to mammalian adaptation. Scientific reports. 2015;5(1):1-7.

12. Cauldwell AV, Long JS, Moncorgé O, et al. Viral determinants of influenza A virus host range. Journal of General Virology. 2014;95(6):1193-1210.

13. Mehle A, Dugan VG, Taubenberger JK, et al. Reassortment and mutation of the avian influenza virus polymerase PA subunit overcome species barriers. Journal of virology. 2012;86(3):1750-1757.

14. Fan S, Deng G, Song J, et al. Two amino acid residues in the matrix protein M1 contribute to the virulence difference of H5N1 avian influenza viruses in mice. Virology. 2009;384(1):28-32.

15. Rosenberg MR, Casarotto MG. Coexistence of two adamantane binding sites in the influenza A M2 ion channel. Proceedings of the National Academy of Sciences. 2010;107(31):13866-13871.

16. Jiao P, Tian G, Li Y, et al. A single-amino-acid substitution in the NS1 protein changes the pathogenicity of H5N1 avian influenza viruses in mice. Journal of virology. 2008;82(3):1146-1154.
