# Supplementary material for: PB1 S524G mutation of wild bird-origin H3N8 influenza A virus enhances virulence and fitness for transmission in mammals
Source: Emerg Microbes Infect. 2021 Jun 6;10(1):1038–51. doi: 10.1080/22221751.2021.1912644 (PMC8183522; doi:10.1080/22221751.2021.1912644)
Supplement: Table_S1.docx [file TEMI_A_1912644_SM6437.docx]

**Table S1.** The genotype of wild bird-origin H3N8 influenza virus in this study. Orange, green, red, and purple blocks represent clade G1, G2, G3, and G4, respectively, in the phylogenetic tree in Figure 1.

| Virus | Group of each gene segment in the phylogenetic tree as shown in Figure 1. | | | | | | | | Genotype |
| --- | --- | --- | --- | --- | --- | --- | --- | --- | --- |
|  | HA | NA | PB2 | PB1 | PA | NP | M | NS |  |
| T222 | 1 | 2 | 1 | 1 | 1 | 2 | 2 | 3 | G1 |
| T51 | 4 | 3 | 1 | 1 | 1 | 3 | 1 | 1 | G2 |
| T75 | 4 | 3 | 1 | 1 | 1 | 3 | 1 | 1 | G2 |
| SH131 | 4 | 3 | 1 | 2 | 2 | 3 | 2 | 3 | G3 |
| SH90-O | 2 | 2 | 2 | 2 | 2 | 2 | 2 | 2 | G4 |
| SH90-N | 2 | 3 | 1 | 1 | 1 | 3 | 1 | 1 | G5 |
| CZ355 | 3 | 3 | 1 | 2 | 3 | 1 | 2 | 1 | G6 |
| CZ322 | 3 | 1 | 1 | 2 | 1 | 1 | 1 | 3 | G7 |
