# Supplementary figures and images for: PB1 S524G mutation of wild bird-origin H3N8 influenza A virus enhances virulence and fitness for transmission in mammals
Source: Emerg Microbes Infect. 2021 Jun 6;10(1):1038–51. doi: 10.1080/22221751.2021.1912644 (PMC8183522; doi:10.1080/22221751.2021.1912644)

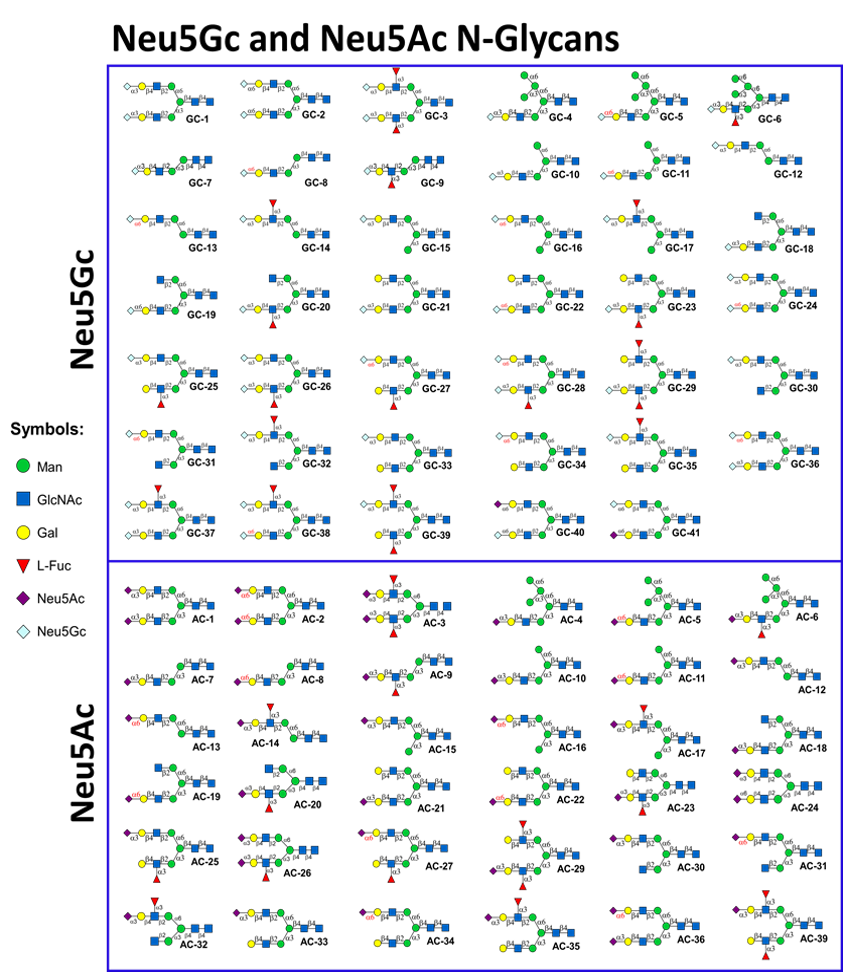


**Figure S5.** The structures of each numbered glycan used in the experiments.

Supplement: Figure_S5.docx [file TEMI_A_1912644_SM6436.docx]
