# Supplementary material for: PB1 S524G mutation of wild bird-origin H3N8 influenza A virus enhances virulence and fitness for transmission in mammals
Source: Emerg Microbes Infect. 2021 Jun 6;10(1):1038–51. doi: 10.1080/22221751.2021.1912644 (PMC8183522; doi:10.1080/22221751.2021.1912644)
Supplement: Figure_S4.docx [file TEMI_A_1912644_SM6435.docx]

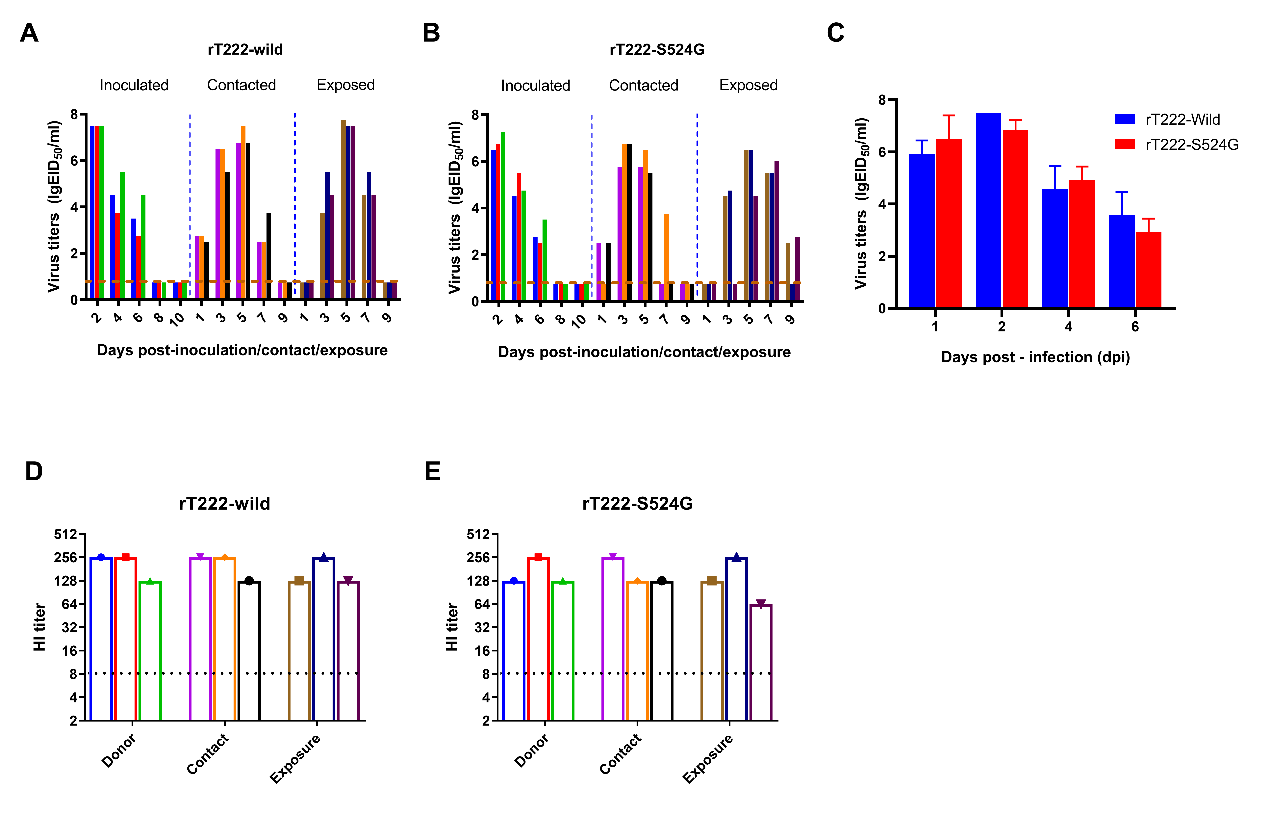


**Figure S4.** Assessment of direct-contact and airborne-contact transmissibility of rT222 and mutant in guinea pigs. The titers of influenza viruses recovered from nasal washes of the virus-inoculated, contact, and exposed animals were shown in A (rT222) and B (rT222-S524G). HI antibody titers of the animals are shown in D (rT222) and E (rT222-S524G). Each column represents individual animals. The dashed lines indicate the lower limit of detection. Average virus titers in the nasal wash of inoculated guinea pigs with the virus at the indicated time are shown in D.
