# Supplementary material for: PB1 S524G mutation of wild bird-origin H3N8 influenza A virus enhances virulence and fitness for transmission in mammals
Source: Emerg Microbes Infect. 2021 Jun 6;10(1):1038–51. doi: 10.1080/22221751.2021.1912644 (PMC8183522; doi:10.1080/22221751.2021.1912644)
Supplement: Figure_S3.docx [file TEMI_A_1912644_SM6434.docx]

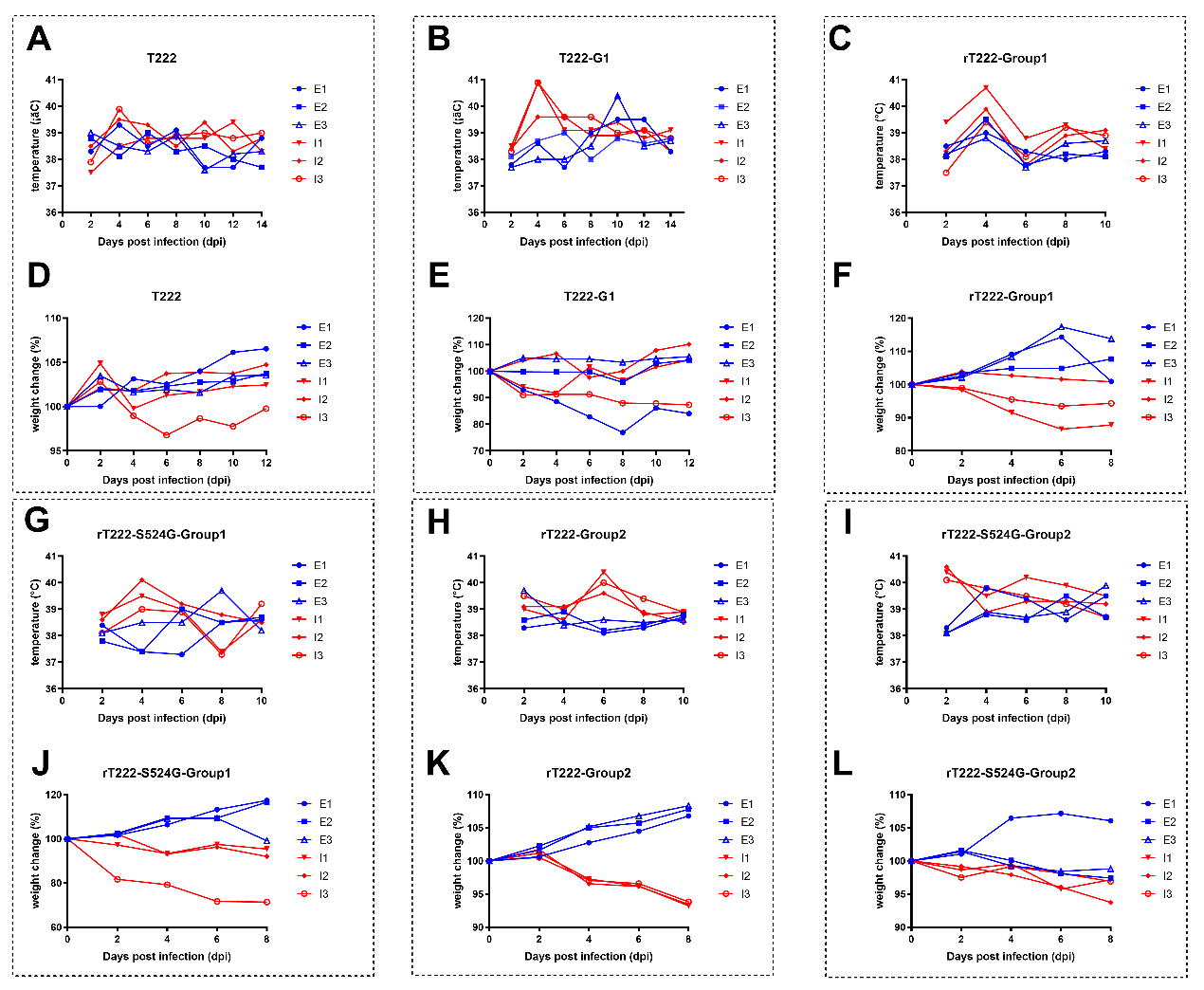


**Figure S3.** The body temperature and weights of ferrets in experiments detailed in Figure 4 and Figure 5. (A and D) T222. (B and E) T222-G1. (C and F) rT222-Group1. (G and J) rT222-S524G-Group1. (H and K) rT222-Group2. (I and L) rT222-S524G-Group2. Each data point represents the weight or body temperature of a single ferret at each time point. I1, I2, and I3 indicate virus-infected animals, respectively; E1, E2, and E3 indicate respiratory droplet exposed animals, respectively. Group 1 and Group 2 represent two parallel repeated experiments.
